# Quantifying microbial DNA in metagenomes improves microbial trait estimation

**DOI:** 10.1093/ismeco/ycae111

**Published:** 2024-09-08

**Authors:** Raphael Eisenhofer, Antton Alberdi, Ben J Woodcroft

**Affiliations:** Centre for Evolutionary Hologenomics, Globe Institute, University of Copenhagen, Copenhagen, Denmark; Centre for Evolutionary Hologenomics, Globe Institute, University of Copenhagen, Copenhagen, Denmark; Centre for Microbiome Research, School of Biomedical Sciences, Translational Research Institute, Brisbane, Queensland University of Technology (QUT), Woolloongabba, Australia

**Keywords:** soil, microbiome, metagenomics, microbiology, microbial ecology, bioinformatics

## Abstract

Shotgun metagenomics is a powerful tool for studying the genomic traits of microbial community members, such as genome size, gene content, etc. While such traits can be used to better understand the ecology and evolution of microbial communities, the accuracy of their estimations can be critically influenced by both known and unknown factors. One factor that can bias trait estimations is the proportion of eukaryotic and viral DNA in a metagenome, as some bioinformatic tools assume that all DNA reads in a metagenome are bacterial or archaeal. Here, we add to a recent debate about the influence of eukaryotic DNA in the estimation of average genome size from a global soil sample dataset using a new bioinformatic tool. Contrary to what was assumed, our reanalysis of this dataset revealed that soil samples can contain a substantial proportion of non-microbial DNA, which severely inflated the original estimates of average genome size. Correcting for this bias significantly improves the statistical support for the negative relationship between average bacterial genome size and soil pH. These results highlight that metagenomes can contain large quantities of non-microbial DNA and that new methods that correct for this can improve microbial trait estimation.

## Main

Average genome size (AGS) is a trait that can be used to better understand microbial ecology and evolution [1]. For example, genome size is thought to reflect environmental and metabolic complexity, with smaller sizes associated with host dependency and reduced metabolic capacity [2, 3]. In a 2023 article, Piton *et al.* [4] observed a relationship between pH and AGS in soil samples, where larger AGSs were observed in lower pH samples. Recently, Osmund *et al.* challenged this relationship, arguing that, since AGS was calculated as the ratio of the number of reads to the number of marker genes detected [5], AGS would be overestimated in soil samples containing eukaryotes. This could lead to systematic biases between different ecosystem types, with Osburn *et al.* [6] arguing that the association between AGS and soil pH reported in the original study may be artefactual. In response, Piton *et al.*, while admitting this methodological limitation, argued that the alternative methodology proposed by Osburn *et al.* could be even more biased, and that soil does not contain substantial amounts of eukaryotic DNA [7]. Both research groups reached consensus on one point: “a perfect estimation of bacterial AGS using soil metagenomes is not yet possible” [7]. While not perfect, we argue that it is now possible to obtain fairly accurate estimations. We recently developed a tool that can account for such biases in AGS estimations [8], and we use it here to address the central uncertainty in this debate.

Our method, SingleM Microbial Fraction (“SMF”), can accurately estimate both the AGS and the fraction of bacterial and archaeal DNA in a metagenome [8]. To begin, the AGS of each species in the GTDB reference database [9] is estimated after adjusting each genome’s size by its estimated completeness and contamination [10]. The AGS of taxons above the species level is inferred from the AGS of the taxons below, e.g. the AGS of a family is calculated as the mean of the AGS of each of its genera. For each metagenome, the read coverage of each taxon is established using SingleM [11]. SingleM sensitively detects nearly all bacteria and archaea in a microbial community, even if they are not included in reference genome databases, by recruiting reads to highly conserved regions of marker genes. The AGS of the entire microbial community is then estimated as the genome size of each taxon present weighted by that taxon’s coverage in the community profile. This method was robust across a range of simulated datasets, and when applied to thousands of publicly available samples, it provided estimates consistent with other forms of validation [8].

Critically, SMF’s AGS estimates are not influenced by eukaryotic DNA—the major concern raised by Osburn *et al.* As part of our recent efforts to showcase the tool, we found that soil contains substantial quantities of eukaryotic DNA (median 31%, *n* = 4160) [8]. This was in stark contrast to the assumption of an “extreme range of 4%–9% eukaryotic base pairs” from Pitton *et al.* [7] (based on Supplementary Note 2 from [7])—an assumption that was critical for their response to Osburn *et al*.

We note that apart from eukaryotic DNA, viral DNA may also influence AGS estimates in the same way. While viral DNA is not likely to be a substantial source of DNA relative to that of eukaryotes, for the remainder of the manuscript we refer to the combination of eukaryotic and viral DNA present in the metagenomes as the “non-microbial fraction.”

**Figure 1:**
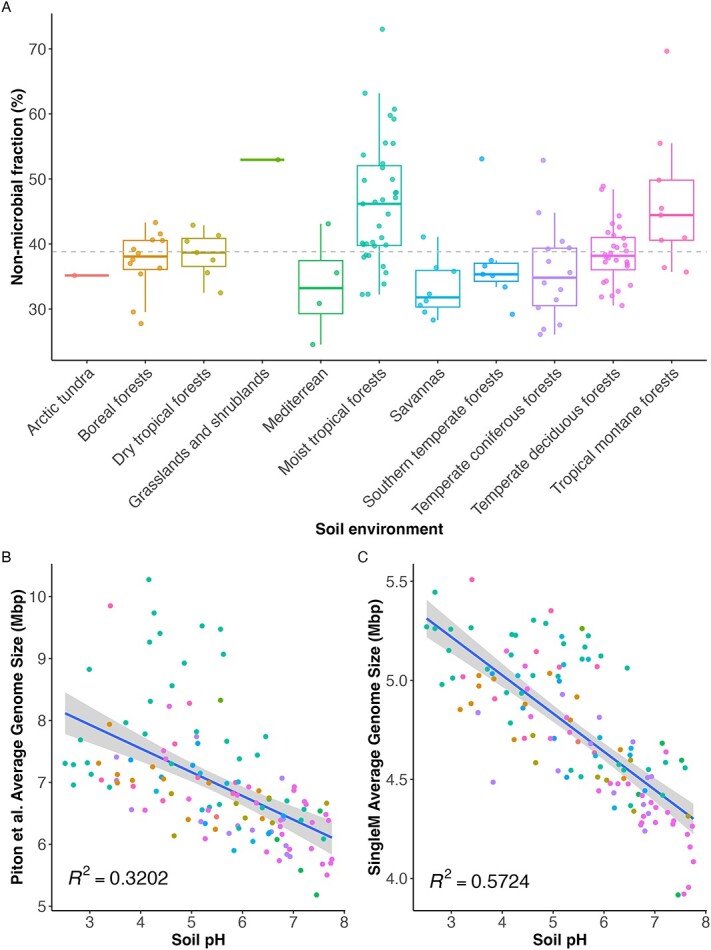
(A) Variation in non-bacterial fraction between environment types. The percentage of non-microbial (eukaryotic and viral) base pairs (*y*-axis) across different soil environment types (*x*-axis. The dashed gray line represents the mean non-microbial fraction for the dataset. (B) The relationship between Piton *et al.*’s AGS and soil pH. (C) The relationship between SMF-corrected AGS and soil pH. *R*^2^ values and best-fit lines are from generalized linear models. *P* values < .0001.

Leveraging SMF, we reappraised Piton *et al.*’s dataset to test whether non-microbial fractions varied across soil types, the extent of AGS overestimation due to this, and whether the original relationship between AGS and soil pH withstood correction. Substantial amounts of non-microbial DNA were predicted (median 38.8%), representing a 9.7- to 4.3-fold underestimation by Piton *et al.*’s extreme range of eukaryotic base pairs [7] (Fig. 1A). We also observed variation in non-microbial DNA quantities both within and between soil environment types (Fig. 1A). While the known species fraction of these samples was low (mean 0.25%), SMF is robust to missing species [8]. Our overall estimate of the mean AGS across all samples was 4.7 Mbp, 31% lower than originally estimated (6.8 Mbp). However, our mean AGS estimate was 36% higher than Osburn *et al.*’s (3 Mbp) estimate. This was likely because their approach only considered reads aligning to bacterial reference genomes, overlooking bacterial sequences not represented in databases [8, 12]. We estimate that, as is common for soil metagenomes [11], only a small fraction of the microbial communities are represented by genomes at the species level (mean 0.5 ± 1.0% s.d.).

Despite the variation of non-microbial DNA observed in Piton *et al.*’s dataset and their overestimation of AGS, we found their original strong negative correlation between AGS and soil pH to be robust (Fig. 1B and C). In fact, correcting for non-microbial DNA abundance led to even stronger statistical support for this relationship (*R*^2^ 0.32 vs. 0.57, Fig. 1C). The relationship was also consistently observed within groups of soils of the same type (see Supplementary Information Fig. S1**)**. Finally, we also found the same correlation in an independent study of forest soils in China cited by Piton *et al*. [13], again with increased statistical support relative to the original study (*R*^2^ 0.42 vs. 0.54, see Supplementary Information Fig. S2).

The application of our newly developed tool contributes substantially to an ongoing scientific discussion that only a few months ago seemed unresolvable, and highlights the complexity and dynamism of the metagenomics research field. Our reappraisal of these data also highlights that non-microbial DNA is currently a severely underestimated component of soil metagenomes. SMF can help identify and control for such biases in future bacterial (and archaeal) community trait estimations not only in soils, but other environments as well. While we have addressed one confounding factor, it is likely that metagenomic analyses still suffer from many unknown biases that will need to be identified and addressed in the near future. Only through critical scrutiny of our data and constructive discussions within the community can we ensure that metagenomics becomes the robust, broadly applicable tool we all aspire it to be.

## Methods

All data and code to reproduce the analyses and figures are available at the following GitHub repository: https://github.com/EisenRa/2024_soil_dark_matter_reply. To estimate the AGSs of samples from Piton *et al.* [4], datasets corresponding to each run accession were sourced from Sandpiper [11]. SingleM v0.17.0 “Archive OTU tables” of each sample were generated by merging those of the corresponding runs, and then re-assigned taxonomy using SingleM “renew” with a GTDB R214 reference metapackage (DOI: 10.5281/zenodo.11123537). The AGS of each sample was then estimated using SingleM “microbial_fraction.” Soil pH values were obtained from https://doi.org/10.6084/m9.figshare.22620025. Data were imported, analysed and visualized in R [14] v4.3.2 using Tidyverse [15]. Generalized linear models were used to correlate AGS to soil pH (glm() function, Gamma distribution).

To estimate the AGS in samples collected by Wang *et al.* [13], reads from bioproject PRJNA986291 were downloaded using Kingfisher (https://github.com/wwood/kingfisher-download). Each run was then analysed using SingleM “pipe” and “microbial_fraction” as above. Measures of pH were those reported as the source data from Wang *et al.*’s Fig. 2. *R*^2^ values were again calculated as above.

We note that reanalysis was undertaken considering bacteria to be the primary constituents of the microbial community, since Archaea were found to be in very low abundance (mean 1.2 ± 0.7% s.d. according to SingleM). Specifically, no attempt was made to separate out the bacterial and archaeal components of the community, and all genome size calculations reported here are those of the total (bacterial and archaeal) microbial community.

## Supplementary Material

Supplementary_information_ycae111

## Data Availability

Reproducible code is available at https://github.com/EisenRa/2024_soil_dark_matter_reply.
