# Supplementary material for: Quantifying microbial DNA in metagenomes improves microbial trait estimation
Source: ISME Commun. 2024 Sep 8;4(1):ycae111. doi: 10.1093/ismeco/ycae111 (PMC11439404; doi:10.1093/ismeco/ycae111)
Supplement: Supplementary_information_ycae111 [file supplementary_information_ycae111.pdf]

Supplementary information

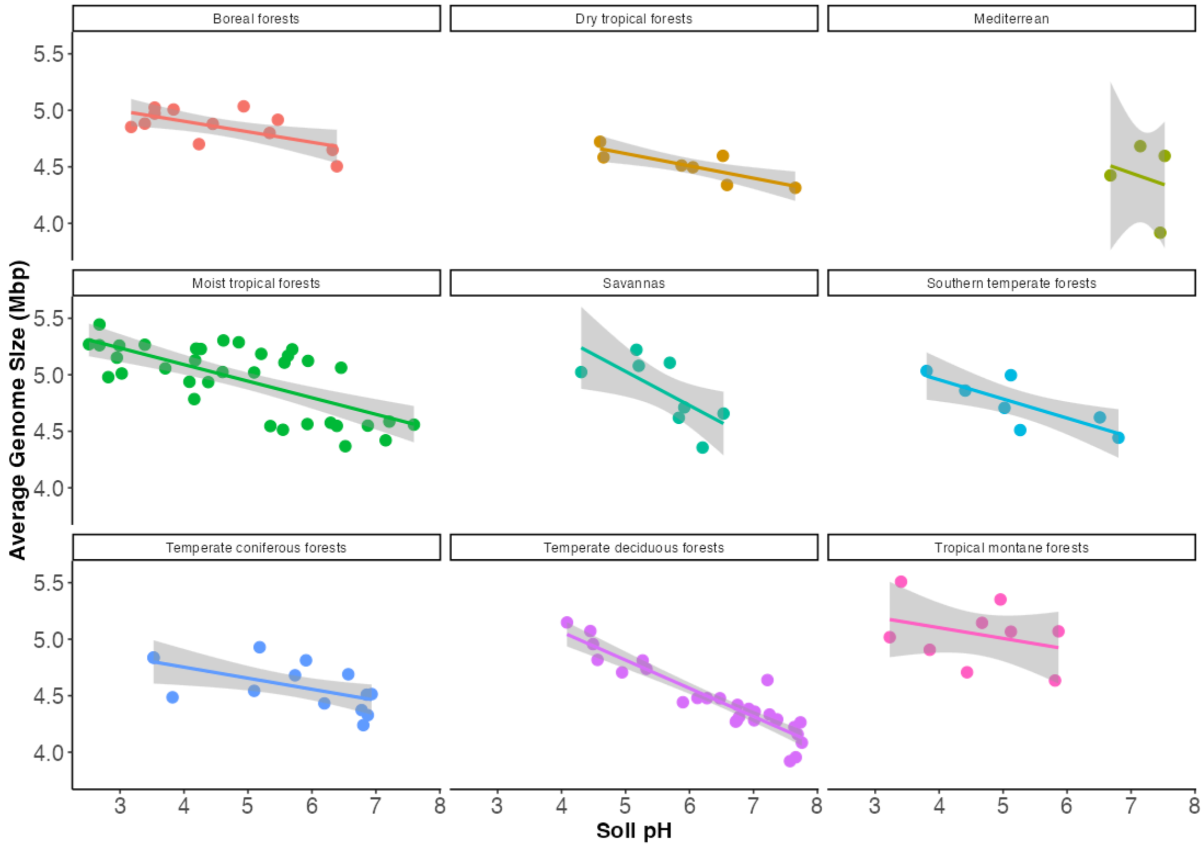

Supplementary Figure 1 | The relationship between corrected AGS and soil pH faceted by soil type. Best-fit lines are from generalised linear models.

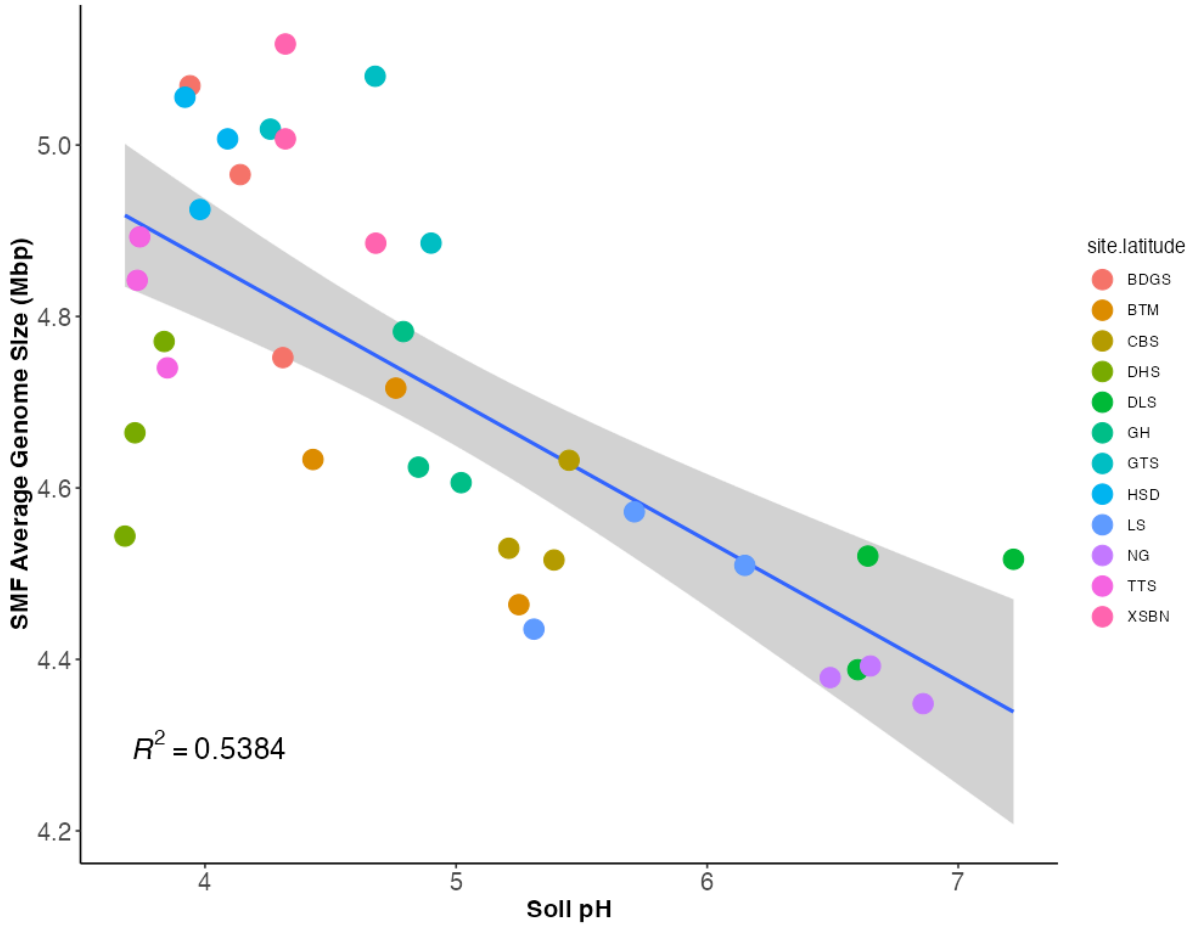

Supplementary Figure 2 | Reanalysis of Wang et al. 2023 soil samples. The relationship between corrected AGS (y-axis) and soil pH (x-axis). R^2 values and best-fit lines are from generalised linear models. $P < 0.0001$.
